# Long-Lasting Remission in De Novo Breast Myeloid Sarcoma Treated with Decitabine and Radiotherapy

**DOI:** 10.3390/diagnostics9030084

**Published:** 2019-07-27

**Authors:** Carla Minoia, Vincenza de Fazio, Giovanni Scognamillo, Anna Scattone, Nicola Maggialetti, Cristina Ferrari, Attilio Guarini

**Affiliations:** 1 Haematology Unit, IRCCS Istituto Tumori “Giovanni Paolo II”, 70124 Bari, Italy; 2 Radiotherapy Unit, IRCCS Istituto Tumori “Giovanni Paolo II”, 70124 Bari, Italy; 3 Pathology Department, IRCCS Istituto Tumori “Giovanni Paolo II”, 70124 Bari, Italy; 4 Radiodiagnostic Unit, Dipartmento di Medicina e Scienze della Salute “Vincenzo Tiberio”, University of Molise, 86100 Campobasso, Italy; 5 D.I.M.-Diagnostic Imaging-Nuclear Medicine, University of Bari “Aldo Moro”, 70124 Bari, Italy

**Keywords:** extramedullary, acute myeloid, leukemia, myeloid, sarcoma, decitabine, breast

## Abstract

Myeloid sarcoma (MS) represents a rare disease with an adverse clinical outcome for patients not candidate to acute myeloid leukemia (AML)-like chemotherapies. Here we present the case of an elderly patient affected by a bilateral breast localization of MS treated with the hypomethylating agent decitabine associated to radiotherapy. The association of the two treatment modalities has allowed an optimal and long-lasting disease control.

## 1. Introduction

Myeloid sarcoma (MS) represents an extra-medullary localization of immature neoplastic myeloid cells, which occurs in 2–9% of patients during the natural history of acute myeloid leukemia (AML). Isolated MS, characterized by the absence of a concomitant AML, is a very rare disease with uncertain incidence and a 5 to 12 month median time to progression into AML [1,2]. The most common sites of MS localization are soft tissues, bones, central nervous system, skin, small intestine, and lymph nodes [2], whereas about 6% of MS involves the breast [3,4,5,6]. The emerging employment of ^18^F-Fluorodeoxyglucose-Positron Emission Tomography/Computed Tomography (^18^F-FDG-PET/CT) has significantly improved the staging of disease as well as the assessment of treatment response [2,7]. While the younger and fitter patients can be candidate for AML-like chemotherapy, for elderly patients a standard therapeutic approach is still lacking [2,8]. Hypomethylating agents, such as azacytidine and decitabine, are currently employed in elderly AML patients, but their role in MS remains unexplored.

Here we describe a peculiar case of MS presenting particular therapeutic features and a long-lasting remission.

## 2. Case Presentation

**Figure 1:**
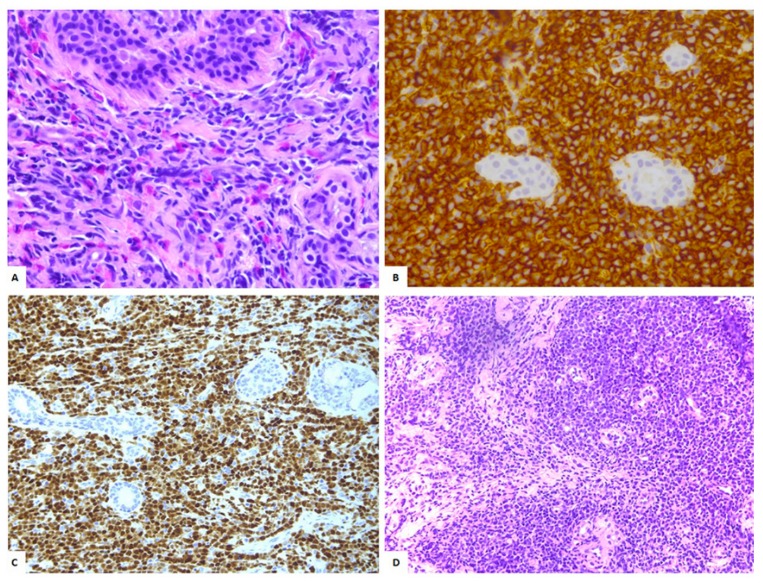
(**A**) Isolated myeloid sarcoma (MS) involving the right breast (40×, H&E): Diffuse infiltration by blasts with occasional eosinophils. (**B**) The neoplastic cells show immunoreactivity for CD34 (40×). (**C**) Anti-TdT immunohistochemical staining (20×). (**D**) Fine-needle biopsy confirmed the presence of a monotonous population of blasts diagnosis of MS involving the left breast (20×, H&E).

A 71 year old female patient, who had relevant comorbidities, including ischemic cardiomyopathy, diabetes, asthmatic chronic bronchitis, and CKD stage-3 chronic renal failure, presented a palpable mass in the left breast. A mammography revealed oval mass measuring 3 cm in axial diameter. She underwent an excisional biopsy, which documented a diffuse and dense infiltration of poorly differentiated, immature neoplastic small- to large-sized cells, with oval to irregular nuclear contours, finely granular chromatin, a single small eosinophilic nucleolus, and scant cytoplasm. Occasional mature eosinophils and eosinophilic precursors were scattered among the neoplastic cells. Immunohistochemical analysis demonstrated that the neoplastic cells were strongly positive for CD34 and TdT with focal reactivity for CD68PGM1, CD117, and myeloperoxidase; the Ki-67 proliferative index was ~60% (Figure 1A–C).

**Figure 2:**
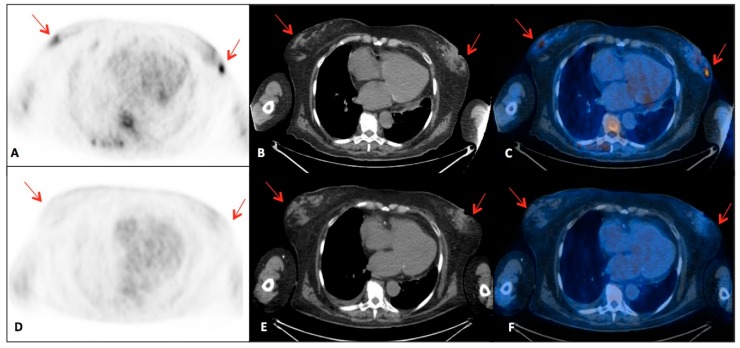
^18^F-FDG-PET/CT examinations performed in a patient affected with breast MS: axial (**A**,**D**) PET, (**B**,**E**) CT and (**C**,**F**) fusion images. (**A**–**C**) Baseline ^18^F-FDG-PET/CT shows bilateral multi-focal breast lesions with increased ^18^F-FDG uptake (SUV max 5.4) (red arrows). (**D**–**F**) Post-radiotherapy ^18^F-FDG-PET/CT demonstrates disappeared of the metabolically active lesions previously described (red arrows) and complete response to therapy.

A diagnosis of MS of the breast was performed. At the time of presentation at our Institute, the patient was asymptomatic and showed normal hemochromocytometric parameters except for a mild anemia. A bone marrow (BM) biopsy showed a tri-linear hematopoietic dysplasia with rare blasts corresponding to 1.2% of CD33^+^/CD117^+^cells at the flowcytometry analysis. Cytogenetic analyses on the BM sample revealed a normal karyotype along with the over-expression of *WT-1,* while no peripheral blasts were detectable. A total-body contrast-enhanced CT documented a mass of solid tissue (3 cm in axial diameter) at the left *corpus mammae* and a similar contralateral lesion (4.5 cm in axial diameter). The bi-laterality of the disease was confirmed by breast magnetic resonance. The PET/CT scan confirmed a bilateral multi-focal ^18^F-FDGuptake, which was particularly intense at the right side (maximum standard uptake value, SUV max 5.4) (Figure 2A–C).

At this level was thus performed a fine-needle biopsy, which confirmed the nature of the disease (Figure 1D).

**Figure 3:**
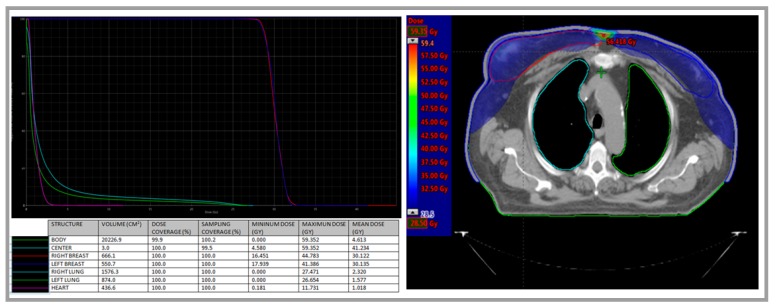
Radiotherapy was performed bilaterally by two opposing fields of 6 MeV photons by Linac D-2300 CD Varian (Palo Alto, CA, USA). The irradiation was administered after placement of a 0.5 cm thick bolus on each breast to limit drastically the “build-up” effect and then insert the skin into the clinical target volume (CTV). A total dose of 30 Gy on each breast was administered. Organ at risk’s dose was reduced to minimize to toxic effects (2.5 Gy to 20% of the right lung; 1.5 Gy to 20% of the left lung; and heart mean dose 1.018 Gy and maximum dose 11.731 Gy).

According to age and comorbidity, since October 2016 the patient was treated with decitabine intravenously at 20 mg/mq for five days (every 28 days). Radiation therapy was also administered between the fourth and the fifth course of decitabine. Radiotherapy was performed bilaterally by two opposing fields of 6 MeV photons by Linac D-2300 CD Varian (Palo Alto, CA, USA), with a daily fractionated dose of 2 Gy for five days a week, until a total dose of 30 Gy on each breast. The irradiation was administered after placement of a 0.5 cm thick bolus on each breast to limit drastically the “build-up” effect and then insert the skin into the clinical target volume (CTV) (Figure 3).

No acute toxicities were reported during radiotherapy. A BM analysis was performed after the sixth course of decitabine and a tri-linear hematopoietic dysplasia with no blasts was confirmed. During the first two cycles of decitabine, the patient presented a grade four anemia and a grade three neutropenia, requiring a treatment delay of seven days at the third cycle. At the eighth cycle she manifested an acute respiratory failure with sub-edema, secondary to a hypertensive crisis and pneumonia. She was treated in the acute phase with C-PAP and medical therapy, including steroids and antibiotics. Pneumonia recovered in one week. After three months from the end of radiotherapy (July 2017), the patient underwent to^18^F-FDG-PET/CT evaluation, which documented a complete metabolic response (Figure 2D–F). Currently, the patient has completed her 22nd cycle of decitabine and maintains the disease remission at 27 months, confirmed by ^18^F-FDG-PET/CT and BM evaluation.

### Statement of Ethics

The research was conducted ethically in accordance with the World Medical Association Declaration of Helsinki. The patient has given the written informed consent to publish the case and images.

## 3. Discussion

This is a rare case of breast MS diagnosed in an elderly patient. Although the majority of MS occur in patients younger than 50 years [4,5], its onset at older age makes the therapeutic approach challenging due to the impaired fitness and comorbidities limiting the administration of intensive chemotherapy. This is a very rare case of bilateral breast involvement, efficiently detected by ^18^F-FDG-PET/CT and confirmed by a bilateral biopsy. Some other reports have documented the sensitivity of the ^18^F-FDG-PET/CT in the detection of extramedullary AML, with particular regards to breast localization. This imaging technique confirmed in our patient its reliability in assessing response to chemotherapy and radiotherapy. Radiation therapy is commonly employed to treat MS and in our patient it was administered bilaterally and defining a peculiar CTV which minimizes the risk of local recurrence but also the toxicity to lung and heart. Some experiences in solid tumors demonstrate an increased radiosensitivity in vitro after exposure to hypomethylating agent, mainly 5-azacytidine [9]. On the other side, in vitro models of breast cancer showed different level of methylation of DNA repair, cell cycle, and apoptosis pathway genes in response to radiation at various doses and time points [10]. At the moment, a synergic role of radiotherapy and decitabine in myeloid sarcoma cells could also be hypothesized.

Decitabine is a cytidine deoxynucleoside analogue that, at low doses, selectively inhibits DNA methyltransferases (DNMMT). This results in gene hypomethylation leading to reactivation of tumor suppressor genes, induction of cellular differentiation or senescence followed by programmed cell death. Our choice to treat the patient with decitabine was mainly driven by clinical parameters as the fitness status and ineligibility to intensive chemotherapy, although no previous reports ever described a breast MS and only a few evidences suggest the use of hypomethylating agents in extramedullary AML. In these reports, moreover, the time to progression towards AML is widely variable and commonly preceded by complete or partial response to treatment, with moderate toxicity [11,12,13,14,15] (Table 1).

Molecular alterations in MS are comparable with AML and mainly affects genes encoding for tyrosine kinases (FLT3, KIT, and KRAS), tumor suppressors (WT1 and TP53), epigenetic modifiers (TET2 and ASXL1), spliceosome proteins (SF3B1 and SRSF2), and transcription factors (RUNX1). But MS was more enriched with mutations of the RTK-RAS pathway genes [16,17]. At the moment, there are no standardized genetic or epigenetic alterations able to predict the response to decitabine and further studies are warranted [18,19].

Our case emphasizes the use of hypomethylating agents, with particular regard to decitabine, as a feasible approach in this rare disease, especially to manage elderly patients. Moreover, the association of decitabine with radiotherapy may result in better long-term response as in our patient who is in complete remission since the 27 months from diagnosis. However, since the treatment with decitabine is not free from potential severe toxicity, especially in patients with comorbidities, analyses of cumulative responses and toxicities in larger cohorts of extramedullary AML patients are needed.

## Figures and Tables

**Table 1:** Reports of patients treated with decitabine for MS.

Reference	Number of Patients	MS Localization	Number of Cycles	Response	OS (Months)
Singh SN et al., 2012 [11]	1	Lymph node/relapse post HSCT	13	CR	26
Modi G et al., 2015 [12]	1	Vagina	4/*ongoing*	PR	4, alive
Gornicec M et al., 2017 [13]	3	Ear	9	Progression to AML	14
		Skin	6	Progression to AML	8
		Skin/relapse	3/*ongoing*	CR	3, alive
Evers D et al., 2018 [14]	1	Pericardium/relapse post HSCT	6 + DLI	PR	6, alive
Castelli A et al., 2018 [15]	1	Skin + BM/relapse post HSCT	8	PR	8
Minoia C et al., 2019	1	Bilateral breast	22/*ongoing*	CR	27, alive

OS, overall survival; HSCT, allogeneic hematopoieicstem cell transplant; CR, complete response; PR, partial response; AML, acute myeloid leukemia; DLI, donor lymphocyte infusions.
